# Migraine headache (MH) classification using machine learning methods with data augmentation

**DOI:** 10.1038/s41598-024-55874-0

**Published:** 2024-03-02

**Authors:** Lal Khan, Moudasra Shahreen, Atika Qazi, Syed Jamil Ahmed Shah, Sabir Hussain, Hsien-Tsung Chang

**Affiliations:** 1 Department of Computer Science, Ibadat International University Islamabad Pakpattan Campus, Pakpattan, Pakistan; 2 Department of Computer Science, Mir Chakar Khan Rind University, Sibi, Pakistan; 3 Centre for Lifelong Learning, Universiti Brunei Darussalam, Bandar Seri Begawan, Brunei Darussalam; 4 Department of Agriculture, Mir Chakar Khan Rind University, Sibi, Pakistan; 5 Bachelor Program in Artificial Intelligence, Chang Gung University, Taoyuan, Taiwan; 6 Department of Computer Science and Information Engineering, Chang Gung University, Taoyuan, Taiwan; 7 Department of Physical Medicine and Rehabilitation, Chang Gung Memorial Hospital, Taoyuan, Taiwan

**Keywords:** Computational biology and bioinformatics, Mathematics and computing, Computational science, Computer science, Information technology

## Abstract

Migraine headache, a prevalent and intricate neurovascular disease, presents significant challenges in its clinical identification. Existing techniques that use subjective pain intensity measures are insufficiently accurate to make a reliable diagnosis. Even though headaches are a common condition with poor diagnostic specificity, they have a significant negative influence on the brain, body, and general human function. In this era of deeply intertwined health and technology, machine learning (ML) has emerged as a crucial force in transforming every aspect of healthcare, utilizing advanced facilities ML has shown groundbreaking achievements related to developing classification and automatic predictors. With this, deep learning models, in particular, have proven effective in solving complex problems spanning computer vision and data analytics. Consequently, the integration of ML in healthcare has become vital, especially in developing countries where limited medical resources and lack of awareness prevail, the urgent need to forecast and categorize migraines using artificial intelligence (AI) becomes even more crucial. By training these models on a publicly available dataset, with and without data augmentation. This study focuses on leveraging state-of-the-art ML algorithms, including support vector machine (SVM), K-nearest neighbors (KNN), random forest (RF), decision tree (DST), and deep neural networks (DNN), to predict and classify various types of migraines. The proposed models with data augmentations were trained to classify seven various types of migraine. The proposed models with data augmentations were trained to classify seven various types of migraine. The revealed results show that DNN, SVM, KNN, DST, and RF achieved an accuracy of 99.66%, 94.60%, 97.10%, 88.20%, and 98.50% respectively with data augmentation highlighting the transformative potential of AI in enhancing migraine diagnosis.

## Introduction

Migraine or Headache is one of the most frequent signs seen by neurologists. It is very common in the general population. A study^1^ revealed that more than 90% of the population suffers from headaches. Patients in China spend 672.7 billion yuan each year^2^ to get medical help in this regard. Although migraine may not pose a serious threat to human life, they can have a significant negative impact on work performance, life quality, and the physiology and psyche of the patient^3^.

Headache itself is an agonizing as well as disabling neurological disease. According to the International Classification of Headache Disorders (ICHD), headache has been broadly categorized into primary headaches (migraine, tension-type headache, and trigeminal autonomic cephalgia or cluster headache), secondary headaches or facial pain, and painful cranial neuropathies^4^.

Primary headache disorders are more prevalent in people globally while the estimated active rate of tension+n-type headache and migraine headache is approximately (40% and 10%) respectively. On the other hand cluster headache which is also known as trigeminal autonomic cephalgia is sporadic compared to tension-type headaches and migraine headaches where their occurrence in the population is nearby 0.1%^5,6^. Migraine headache with severity is a very common neurological disease with an incidence of 1 year approximately 16% in the overall population. It is the second highest prominent brain condition in the world as well as causes higher impairment than any other neurological ailments together^7^. Migraines are categorized into; migraines with aura, migraines without aura, and chronic migraines. Around 25% individuals are those who experience aura migraines. The symptoms of aura migraine include the gradual appearance of visual, speech, and other central nervous-related signs. A migraine with an aura lasts an hour^8^. , mainly appearances in migraines without aura have unilateral, pulsating, moderate to severe pain, annoying by or causing to avoid of daily physical activity. When the headache is treated not well it lasts from 4 to 72 h and has signs associated with nausea, vomiting, phonophobia, and photophobia^9^. However, chronic migraine refers to a migraine recurrence whereby an individual has at least 15 headache days per month, along with at least eight completely established migraine days per month^10^. Human beings in the current scenario live in a digital environment, where everything as well as their lives are connected with data sources and are inscribed digitally^11^.

In the medical world, each disease is distinguished from the others by its symptoms. In migraine sufferers, the following symptoms appear Vomiting and Nausea, Increased urination, Lethargy (lack of energy), Phonophobia (Noise), Photobhobia (Sensitivity to light), and Throbbing headache.

The disease of migraine is abandoned. A patient’s migraine headache (MH) begins as a result of some triggers. An event or behavior that you experience or engage in that seems to cause a migraine episode is referred to as a trigger. There are some migraine triggers such as Stress, Drinks (coffee), Medication, Sleep changes, Changes in the weather, and Hormonal changes in women.

Traditional migraine classification and prediction machines such as Magnetic Resonance imaging (MRI), Positron emission tomography (PET), and computed tomography (CT) scans are very expensive. Additionally, very highly experienced medical doctors are required to advise migraine patients. Especially, people living in developing countries can’t afford it financially and there is also a dearth of resources for migraine classification and prediction. Therefore, an automatic affordable, accessible approach is required for migraine classification and prediction. Fortunately, machine learning algorithms show state-of-the-art performance in tasks such as text classification^12–15^ speech recognition^16–19^, and many health-related automatic disease classifications and predictions^20,21^.

The majority of the existing models were relatively trained on tiny data sets, therefore despite significant efforts being made for ML-based migraine classification tasks, their performance is not up to par. Furthermore, real patient data sets are not used by the existing models. In contrast with others, we used a slightly larger data set with data augmentation for training our suggested DNN model. The performance of our suggested model outperforms that of earlier studies since the corpus size was expanded utilizing the data augmentation technique. The suggested models are also trained using data from actual patients. As a result, the models we’ve suggested paint a true picture of this migraine classification. The primary contributions of this investigation are as follows:

- The key contribution of this paper is the investigation of various machine learning algorithms for classifying different types of migraines. The study explores the use of support vector machine (SVM), K-nearest neighbors (KNN), random forest (RF), decision tree (DST), and deep neural network (DNN) algorithms trained on a publicly available dataset. The study also investigates the impact of data augmentation on the performance of these algorithms. The results of the study show that the proposed models with data augmentation achieve high accuracy in classifying the different types of migraines, with the DNN model achieving the highest accuracy of 99.66%. The study shows that these machine learning algorithms could be effective tools for predicting and classifying migraine in patients, particularly in underdeveloped countries where there is a lack of medical instruments, staff, and doctors. Overall, this paper contributes to the growing body of literature on the use of machine learning in healthcare and demonstrates the potential of these techniques for improving diagnosis and treatment of migraine.
- To use and find the effectiveness of data augmentation techniques, and train and test proposed models on actual patient data.
- We used a data augmentation technique to expand the data set, which is another addition. It will be made public for further investigation. Finally, we provide a Python code for the Migraine classification framework. We believe that the enhanced corpus for migraine and model library will be useful in fostering research in migraine classification in underdeveloped countries.
- Another key contribution is to compare and identify the best classifier among the applied machine learning algorithms after data augmentation. A set of machine learning models such as SVM, Random Forest, KNN, DST, and DNN were implemented to predict and classify migraine.

The rest of the paper is structured as follows: the related research is described in "Related work" section, and the proposed methodology and corpus are explained in "Materiels and method"Section. The experiments and their revealed findings are described in "Experiments" section IV. Portion V, explains the conclusion and future work.

## Related work

Although Machine learning exhibits huge success in health care data related computer-aided diagnosis, image certification, image classification, image-guided medical aid, data repository acquisition, multidimensional image integration, and medical image dissection still there is a lot of work that needs to be done chen2021ethical. Inside the medical industry, ML has undoubtedly had relatively little social influence. The answer to lowering the rising cost of treatment and fostering better clinician-patient dialogue is found in ML. Frequent health-related applications of ML techniques comprise helping physicians find numerous patient-specific medications and treatments, as well as helping patients choose when and whether they need to schedule follow-up consultations^22^. There is presently a vast amount of content available in the medical industry. It includes EMRs which are made up of data, both structured and unstructured. Structured clinical data is easy to evaluate in a database and will include several figures and classifications in addition to patient weights but also perhaps general signs like headaches and stomachaches^23^. The majority of clinical data consists of unstructured data that is spread throughout a wide range of annotations, pictures, podcasts, assessments, and exit statements. A discussion between a provider and a patient is very difficult to quantify and evaluate since it is very individualized and might go in a lot of different ways^24^. Such ML algorithms help locate complex correlations in a wealth of enormous data. Healthcare procedures, notably ones that depend on cutting-edge genomes and proteomics analysis, are exceptionally compatible with this technology. It is frequently employed in the diagnosis and monitoring of many illnesses. ML algorithms will provide better therapeutic strategies for individuals in clinical sectors by making recommendations for building beneficial medical systems^25^. Inside the fields of machine learning and data science, several classification methods have been suggested. Foremost extensively utilized techniques across a range of application areas are outlined in the sections that follow; such as Naïve Bayes, Linear Discriminant Analysis (LDA), Logistic regression (LR), K-nearest neighbors (KNN), decision tree (DST), random forest (RF), Fuzzy logic, support vector machine (SVM), and Classification and Regression tree (CART)^26^.

In Study^27^, authors used five supervised machine learning techniques to classify migraine disease based on the subject symptoms. A Weka data mining tool was used for the implementation and classification. According to the results, the Naïve Bayes model was the best-suited and simplest algorithm out of the chosen models.

EGG signals were used in study^28^, to detect and classify migraine types. Computer-aided diagnostic (CAD) system that utilized deep learning models such as VGG16, ResNET101, and DenesetNet121 to classify migraines. In another study^29^, electroencephalography (EEG) was used to support proficient decisions in the automatic detection of migraine. The dataset of EGG signals consists of 18 migraine and 21 healthy volunteers. The revealed results showed that the Bi-LSTM algorithm with 128 channels achieved the highest accuracy of 95.99% when compared with the other models (support vector machine, linear discriminant analysis, and random forest). In another study^30^, authors used clinical data annotated by domain experts of 400 patients. Initially, authors collected symptom-based data, then they identified and selected the 24 most related variables, after related variables selections artificial neural network (ANN), and other traditional ML models were used to classify migraine. The artificial neural network model achieved the highest accuracy of 97% for migraine classification tasks as compared to other used models such as SVM, LR, decision tree, and nearest neighbor.

In Study^31^, somatosensory evoked potential features were built in time and frequency domains for migraine classification using machine learning algorithms including (support vector machines, random forest, K-nearest neighbors, extreme gradient boosting trees, linear discriminant analysis multilayer perceptron, and logistic regression). They were able to achieve over 88% accuracy in interictal or migraine ictal versus healthy detection.

The inception module-based CNN Approach showed improvement over 86.18% when compared with the traditional support vector machine which gained an accuracy of 83.67% to discriminate healthy and migraineur controls along with the two subtypes of headaches^32^. In Study^33^, a feature selection method was introduced to improve the classification of the migraine group. With the naïve Bayes, SVM, and Adaboost classifiers the performance was improved from 67 to 93%, 90 to 95%, and 93 to 94% respectively. Similarly, another technique was proposed by^34^ for early diagnosis of migraine disease by using EGG signals. ANN reached the highest accuracy of 88% among SVM and logistic regression. During the mental arithmetic task (MAT), functional near-infrared spectroscopy was used to observe hemoglobin change in the prefrontal cortex (PFC). The specificity and sensitivity of that model showed 75% and 100% for Chronic migraine (CM) respectively. Similarly, the model achieved 100% specificity and 75% sensitivity for Medication-overuse headache (MOH). According to the results, fNIRS is more feasible for migraine classification when combined with machine learning^35^.

In Study^20^, the primary focus of the authors was to design and implement a decision support system for diagnosing tension-type and migraine headaches using machine learning. The logistic regression model achieved the best results with an accuracy of 0.84 out of other models such as gradient boosting algorithms and random forest.

Data mining techniques play a vital role in the field of medicine. In study^36^, Data mining classification techniques were used such as Naïve, KNN, SVM, and random forest. Among these, all Naïve bays was the best classifier with a precision of 0.905 and an accuracy of 0.475. A realistic monitoring scenario was proposed for monitoring hemodynamic variables from real patients in which a wireless body sensor network (WBSN) was used. N4SID models were developed which provide a low rate of false positives and average forecast windows of 47 min^37^. A machine learning technique was used to differentiate between healthy and migraine patients with the combination of three functional measures of rs-fMRI^38^.

Ufuk et al.^39^ proposed DNN for diagnosing migraine and achieved 95% accuracy. The authors used 8 attributes and diagnosed 3 types of migraine (migraine with aura, c, and migraine without aura). Ferroni^40^ proposed DSS for diagnosing migraine due to medication overuse and achieved 82% accuracy. Another study^41^ proposed DSS for diagnosing primary headaches and achieved 80% accuracy. The authors compared four different machine learning techniques as Bagging, Navie-Bayes, Boosting, and Random Forest. Rober keight^31^ proposed DST for diagnosing types of primary headaches using 9 types of Machine Learning classifiers and achieved 95% accuracy. Hao Yang^42^ proposed CNN for MRI-based classification of migraine and achieved 99% accuracy. Akben^43^ implemented ANN for diagnosing migraine and achieved 83.3% accuracy. Akben^44^ deployed an SVM classifier for diagnosing migraine and achieved 85% accuracy. Subasi^45^ used various versions of Random Forest for diagnosing migraine and achieved 85.95% accuracy. De la Hoz^46^ used ANN for diagnosing migraine and achieved 88% accuracy. Yolanda Garcia^33^ suggested features selection for diagnosing migraine and achieved 90% accuracy. Recently, scholars have been increasingly focusing on utilizing MRI and fMRI images for the detection and classification of migraine headaches^47–49^.

**Figure 1:**
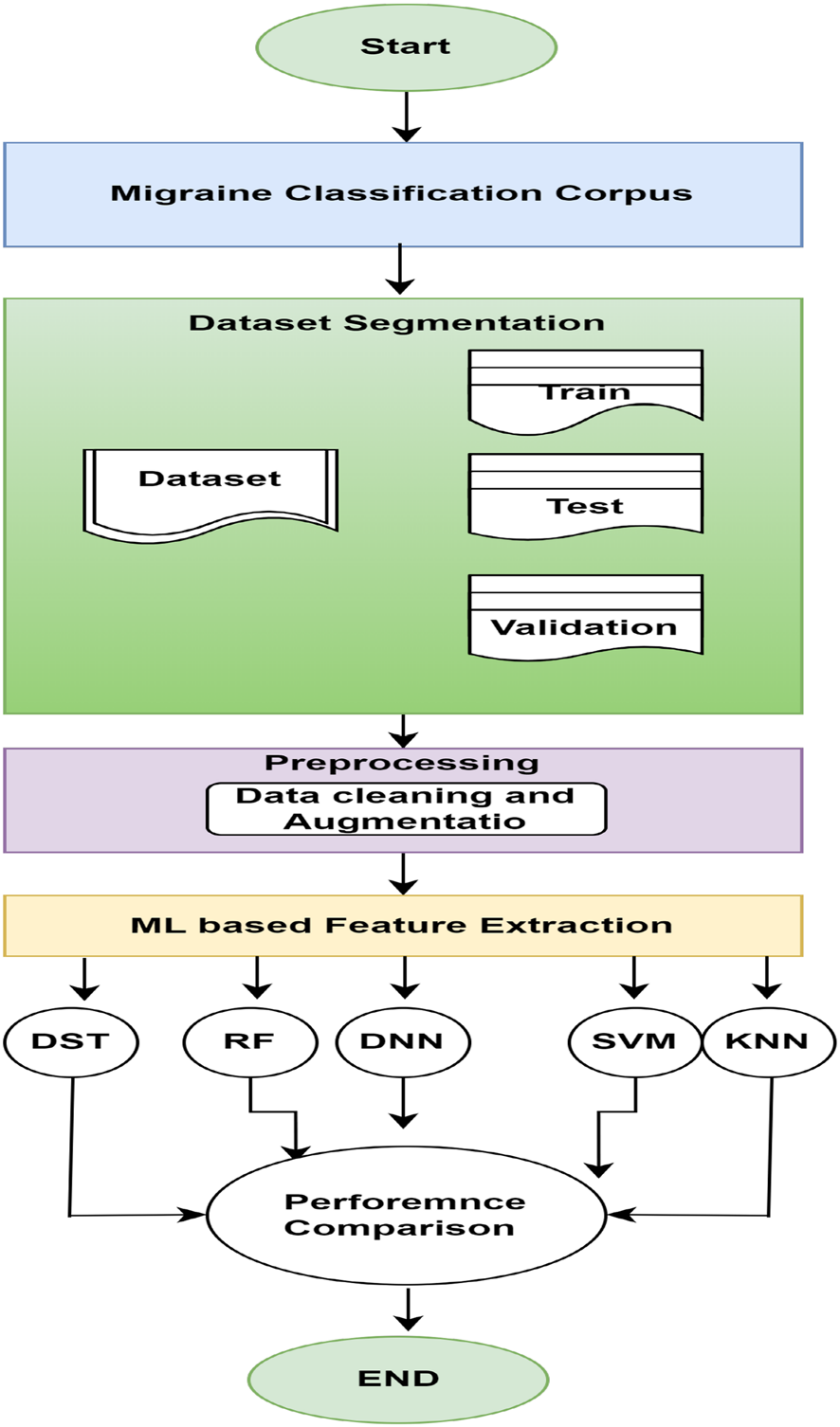
Overall flow of proposed system for migraine classification.

**Figure 2:**
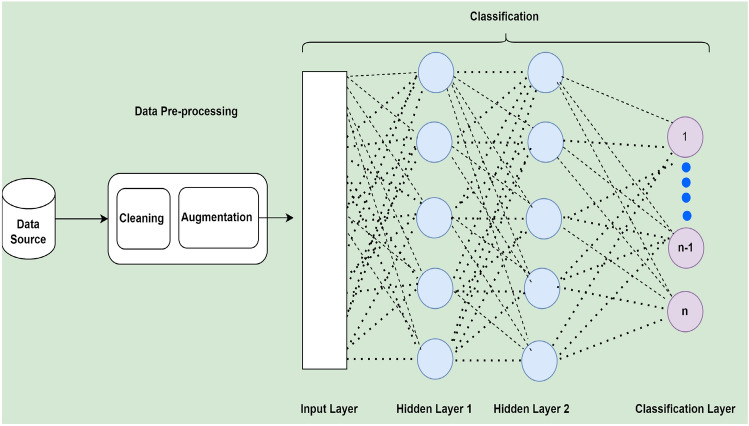
Basic system architecture of deep neural network used for migraine classification.

**Figure 3:**
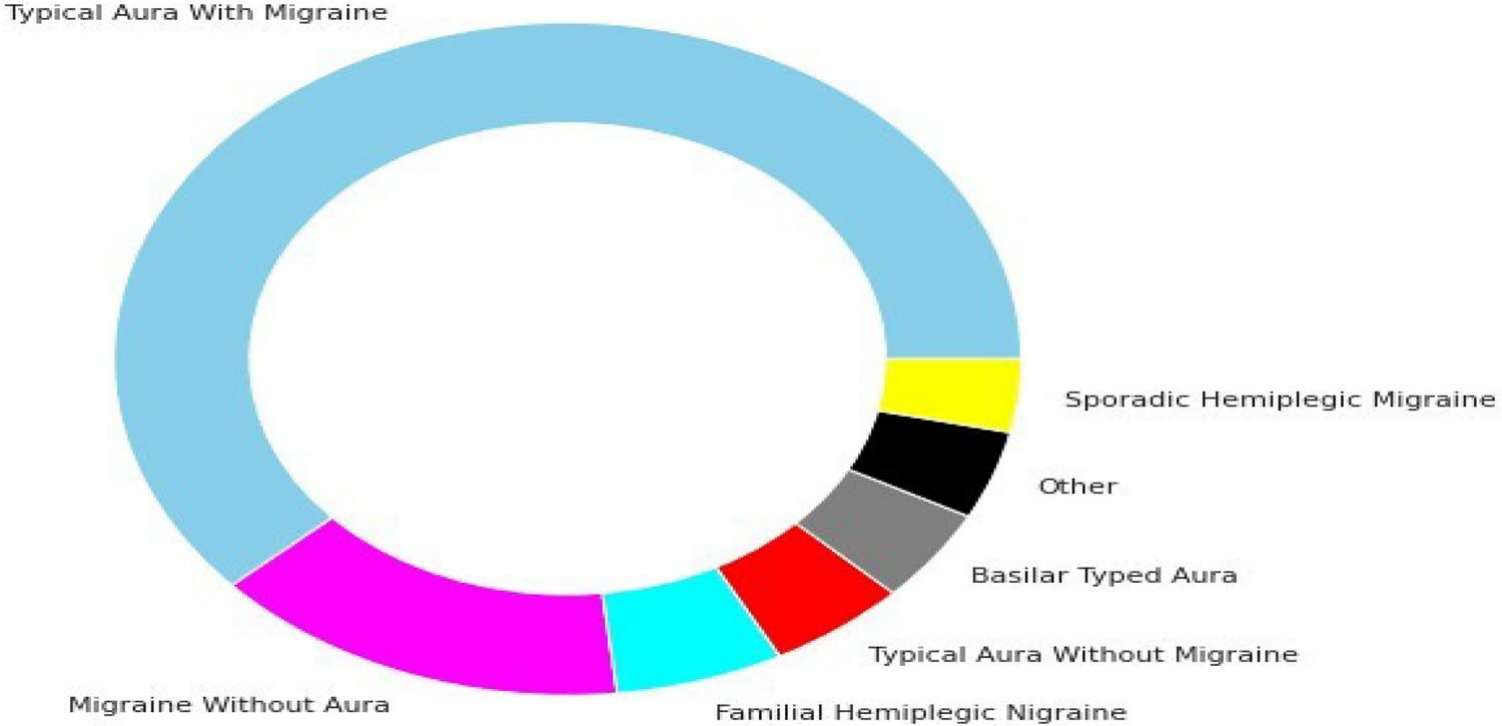
Class imbalance before data augmentation.

**Figure 4:**
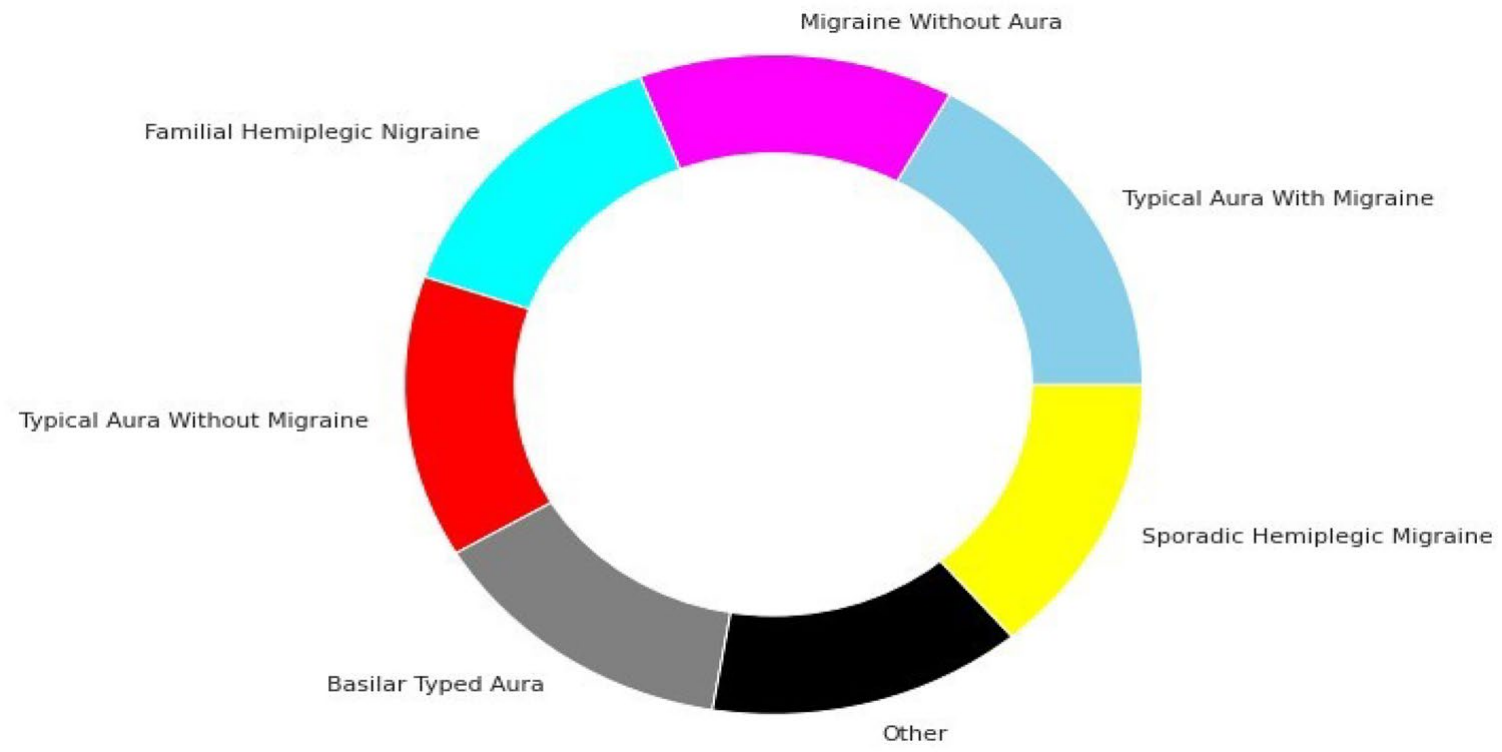
Class balance after data augmentation.

**Figure 5:**
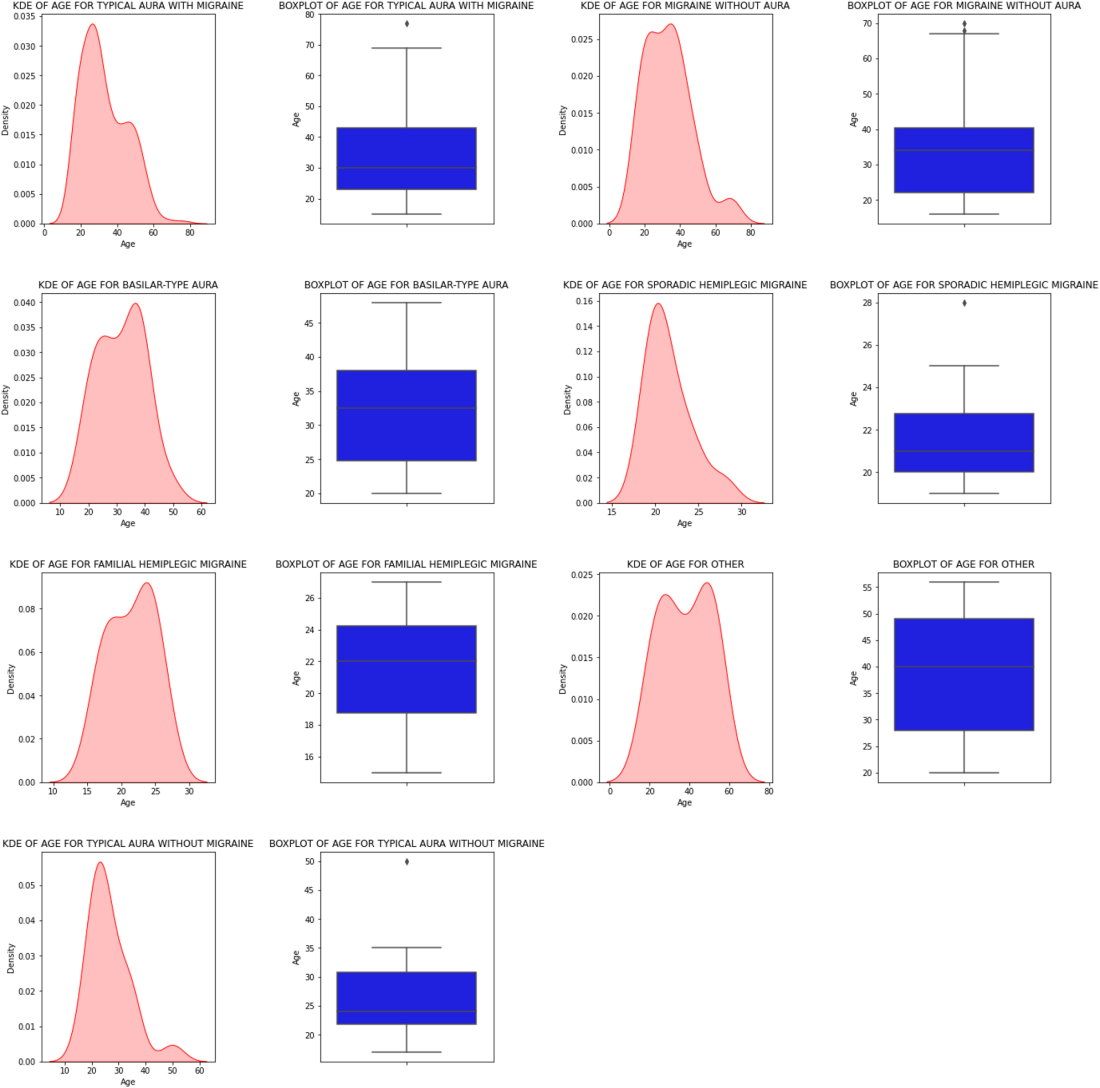
Age-wise distribution of patients in the data frame.

## Materiels and method

Processing data requires much time and computing resources, even using elementary methods such as machine learning or deep learning. Therefore, collecting relevant insights requires a robust machine-learning network. Creating a sophisticated ML framework is also tricky. Personalization is made possible by modifying the parameters of a machine learning classifier. In this paper, the models were trained using various ML algorithms. Moreover, the parameters of the ML algorithms were modified and tailored to the input dataset for enhanced classification. Machine learning algorithms that have been used in this study as shown in Fig. 1 are the Support vector machine, K nearest neighbor, decision tree, random forest, and deep neural network.

### Pre-processing

It is crucial to pre-process the data before supplying it to machine learning models to get better results. Noisy data is eliminated, inconsistent data is made consistent, and error identification and data translation into numerical variables were carried out on the experimented migraine corpus. and data augmentation is chosen to increase the volume of the corpus during the pre-processing stage. Noise has been eliminated by transforming the object data and removing irregular patterns, such as atypical data, poor typing, blank, incomplete, and inconsistent data. Data augmentation is a method to increase the volume of data by making new data points to current data. Synthetic Minority Oversampling Technique (SMOTE)^50^ is a data augmentation technique used to increase the data size and minimize the unbalancing problem. SMOTE adds synthetic minority class examples to the original data set. We used the SMOTE augmentation technique to achieve a balanced data set.

### Dataset

Four hundred people’s medical histories were reviewed, all of whom had been diagnosed with one of many diseases linked to migraines. Expert medical personnel from the Hospital Materno Infantil de Soledad collected data. The patient’s name, address, insurance company, primary care physician, symptoms, age, patient ID, treatment plan, etc were all stored in the dataset. In contrast, this investigation puts emphasis only on clinical presentation. A need for personally identifiable information about patients was not present.

Our used dataset initially consisted of 400 patient records with 24 attributes. After the data augmentation process, the corpus size increased to 1447 patient records. Training and testing data were separated from the dataset. Out of a total of 1447, 1157 records made up the training set, and the rest were used for testing. The dataset was significantly unbalanced before data augmentation, as shown in Fig. 3. However, Fig. 4 demonstrates that the dataset is perfectly balanced after data augmentation. The Fig. 5 represents the age-wise distribution of patients. The proposed dataset comprised 7 migraine classes named: (a) Typical aura with migraine, (b) Migraine without aura, (c) Typical aura without migraine, (d) Familial hemiplegic migraine, (e) Sporadic hemiplegic migraine, (f) Basilar-type aura, and (d) Other.

### Classification models

After applying basic pre-processing and data augmentation techniques, a set of machine learning classifiers such as SVM, KNN, DT RF, and the deep neural network (DNN) models have experimented with the migraine classification corpus. Figure 2 shows the basic architecture of DDN. A total of four layers such as inputs, two hidden layers, and classification layers are designed for the migraine classification task.

## Experiments

This section elaborates on an extensive set of experiments conducted on the dataset to validate the effectiveness of data augmentations

### Hyper-parameters

The Table 1 describes the parameters that were used during DNN experimentation.

**Table 1:** Hyper-parameter used against various models.

Model	Hyper-parameter	Value
multirowDNN	Number of epochs	100
	Activation function	relu
	Optimizer	Adam
	Model	Sequential (first layer)
	Number of neurons at first dense layer	512
	Hidden layer	2
	Classification function	softmax
	Loss function	categorical-cross entropy
SVM	Kernel	Linear
	Class	class sklearn.svm.SVC
	Regularization parameters C	1
	probability	True
KNN	Neighbors range	(1,15,1)
	weights	Uniform
	Metric distance	Euclidean

### Discussion

**Figure 6:**
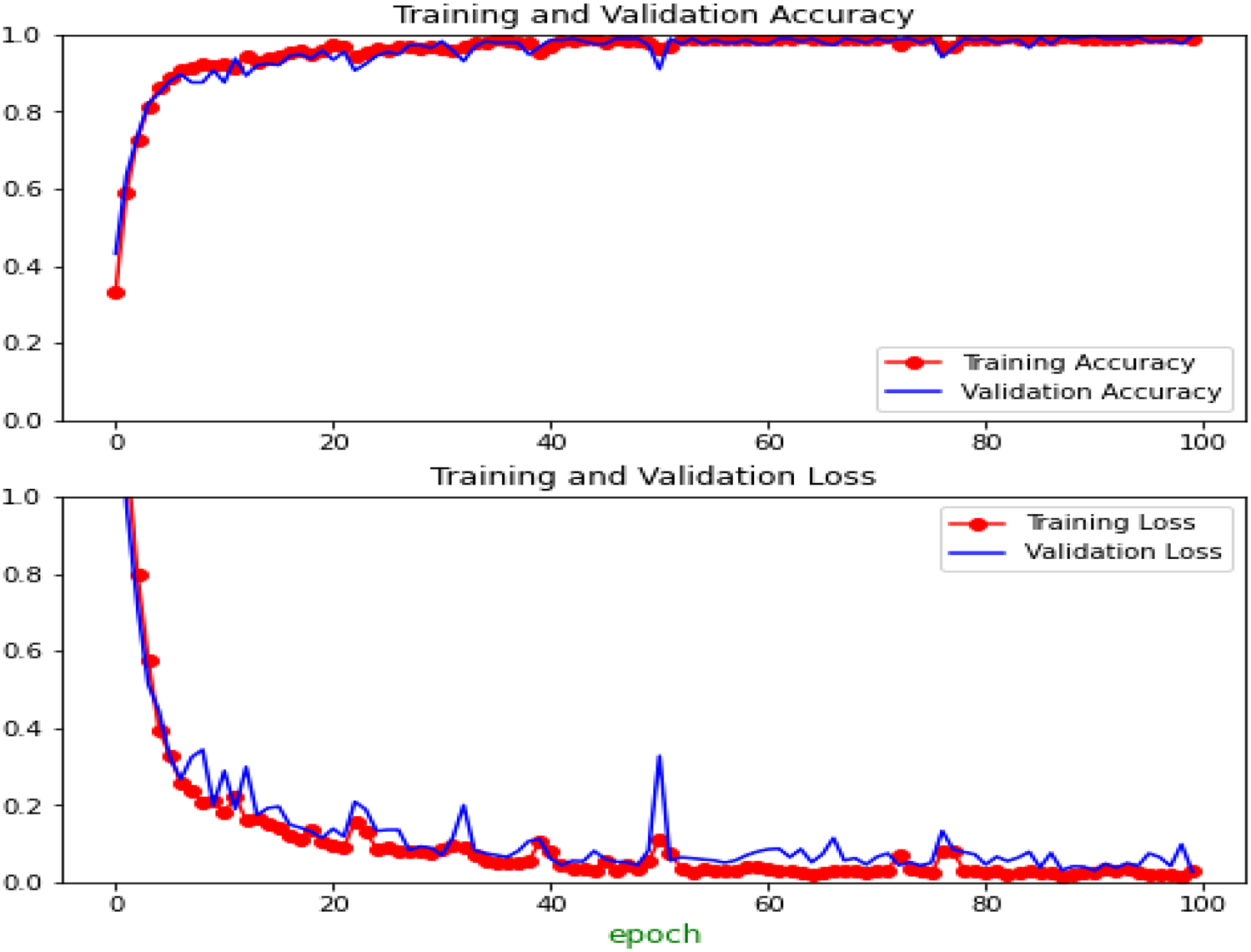
Accuracy and loss graph of deep neural network with data augmentation.

A corpus of 400 clinical records of patients with varied pathologies related to migraines was initially employed in the suggested study paper. After using the data augmentation technique the number of patient records was increased to 1447. Initial data were collected by skilled medical staff at the Hospital Materno Infantil de Soledad, Colombia in early 2013. The experiments were implemented before and after data augmentations. Various ML classifiers such as KNN, SVM, RF, DST, and DNN were used before and after data augmentation. The experimented dataset comprises patient ID, HealthCare ID, physician ID, migraine symptoms, identified or diagnosed disease, and treatment. However, our proposed study used only diagnosed disease and migraine symptoms. Identifiable data were not used in this paper. A total of 23 variables such as age, family background, dizziness, vomiting, etc. were identified and selected related to symptoms that patients have during headaches. Additionally, one variable is identified and selected which is related to the diagnosis of migraine type. The value of symptoms-related variables can be, dizziness, vomiting, etc. On the other hand, the value of diagnosis-related variable represents migraine “Type” labeled by the treating physician based on medical history and symptoms of the patients, probably with one of the migraine types presented in Fig. 4.

The revealed results were authenticated and created on the type of migraine annotated by the Human domain expert and spending the particular measurement made through the classification experiment revealed ML algorithms and deep neural network (DNN). The training and validation accuracy of the DNN model is presented in Fig. 6. The highest accuracy was achieved by a deep neural network implemented with 512 hidden neurons at a dense layer, which gained an accuracy of 99.6% with data augmentation. Table 2 demonstrates that the classification performance achieved by our proposed DNN+ Data augmentation model for headache migraines matched with the classification made in 97% of the 80 test instances made by the domain expert (treating physician). Due to its deep nature, the deep neural network achieves the maximum accuracy in comparison with traditional ML models such as KNN, SVM, RF, and DST. As already established deep learning models are hungry for data and give better results when these models are trained considerably with bigger datasets.

In Table 3, the results of existing work for migraine classification are equated with the accuracy of the classification produced by our proposed model with data augmentation. All other existing models outperformed the ML models and DNN with the Data Augmentation strategies we provided. The data augmentation techniques proved very effective since the model’s performance increased by 2%.

**Table 2:** Classification Report of all used algorithms with and without data augmentation technique.

Model	Precision	Recall	F1-Score	Accuracy (%)
SVM	92.00	94.00	92.98	93.10
SVM + Data Augmentation	93.20	95.00	94.09	94.60
KNN	91.00	100.	95.28	95.70
kNN + Data Augmentation	100.00	93.00	96.37	97.10
DST	78.00	97.00	86.40	87.10
DST + Data Augmentation	80.15	97.500	87.97	88.20
RF	78.50	97.00	86.77	87.40
RF + Data Augmentation	80.50	97.50	88.18	88.50
DNN	97.00	98.00	97.49	97.50
DNN + Data Augmentation	99.00	98.50	98.74	99.66

**Table 3:** Classification Report of all used algorithms with and without data augmentation technique.

Study	Used model and data number	Pre-processing	Accuracy (%)
^51^	RF was implemented to diagnose primary headache and only 260 data instances were used	–	81
^30^	400 clinical records were classified using ANN	minimizing noise, scaling the large-sized object data, and removing inconsistent data	9
^45^	RF was proposed to detect migraine by using photic stimulation	Cleaning, correcting typing mistakes	85.95
^52^	LDA was proposed to detect migraine with Aura	Cleaning, correcting typing mistakes	97.44
^53^	XGBoost model was used to classify migraine type	–	80.71
Proposed study	SVM + Data augmentation	Cleaning, noise removing and Data Augmentation	94.60
Proposed study	KNN + Data augmentation	Cleaning, noise removing and Data Augmentation	97.10
Proposed study	DST + Data augmentation	Cleaning, noise removing and Data Augmentation	88.20
Proposed study	RF + Data augmentation	Cleaning, noise removing and Data Augmentation	88.50
Proposed study	DNN+ Data augmentation	Cleaning, noise removing and Data Augmentation	99.66

Furthermore, in this research work, we applied various machine learning models and preprocessing techniques using Panda’s library for cleaning and removing outliers from the migraine dataset. Additionally, we have applied various SVC kernels and preprocessing techniques for better performance. For model evaluation, we also used different performance metrics, such as the classification report, and the confusion matrix. However, the deep neural network outclassed all the traditional machine learning classifiers and got an accuracy score of 99.66% on the migraine dataset.

Over the past few years, there has been a proliferation of various automatic classification techniques based on machine learning. These include ANN, DT, logistic regression, Bayesian classifiers, nearest neighbors, support vector machines (SVMs), and multiple discriminant analysis^54,55^. Table3 lists the accuracy-based migraine-type classification results from previous studies utilizing accuracy as an evaluation measure. In comparison to other earlier studies, our suggested data augmentation strategy using a deep neural network model with two hidden layers and 512 neurons achieved the best accuracy of 99.66%.

## Conclusion

Migraine is a very common and complex neurovascular disorder. Medical Doctors commonly use scales to identify and classify migraine into their types. As it’s a common disease and every second/third person is suffering from migraine the ratio between patients and medical doctors is too high. On the other hand, underdeveloped or developing countries have a shortage of well-trained doctors, medical staff, and medical instruments. Therefore, we need to utilize machine learning models to classify and predict the migraine. Because machine learning models showed state-of-the-art performances in task-related text classification and automatic prediction. In this study, we implement four traditional machine learning algorithms such as SVM, KNN, Random Forest, and DST. We used various performance evaluation techniques such as Confusion Matrix, Accuracy, and F1-measures to compare the revealed results of proposed algorithms with existing studies. After preprocessing steps, the proposed machine learning algorithms were trained on the publicly available corpus to classify migraine into its basic seven types. The revealed results show that The DNN beats other applied traditional models quite comprehensively.

## Implications

The findings from this research on migraine diagnosis using machine learning (ML) have crucial implications for policymakers in the healthcare sector. Here can write some such as

*Implications:* Policymakers stress the necessity for extensive awareness campaigns to overcome the difficulties in migraine diagnosis. Due to the limitations of subjective pain intensity ratings, migraine headache, a common and complex neurovascular disorder, presents considerable problems in clinical identification. It is critical to raise awareness of headaches among the general population because they have a profound influence on people’s brains, bodies, and overall functioning and have low diagnostic specificity.

policymakers should give top priority to creating and implementing awareness campaigns that inform the public and healthcare professionals about the value of early detection, accurate diagnosis, and AI-based remedies. By raising awareness, policymakers can encourage people to seek prompt medical attention, make it easier to incorporate ML into healthcare systems, and eventually improve migraine management as a whole.

*For researchers:* Overall, this study demonstrates how machine learning models may be used to solve the difficulties in migraine diagnosis and categorization, particularly in areas with few medical resources.

*Future work:* Future developments can improve accuracy and change the migraine therapy landscape by utilizing cutting-edge algorithms and growing datasets.

Although, the machine learning algorithms performed well still there is enough room to improve the performance in the future by using the latest transformer-based algorithms such as Bidirectional Encoder Representations from Transformer (BERT). There is a dearth of publicly available datasets for migraine classification therefore in the future, we plan to build a new comparatively big dataset for migraine classification to achieve higher accuracy.

## Data Availability

Migraine classification dataset is publically available on the internet on various platforms such as Kaggle. The link to dataset is: https://www.kaggle.com/datasets/weinoose/migraine-classification.
